# Adverse events in different administration routes of Edaravone: A pharmacovigilance study based on the FDA adverse event reporting system

**DOI:** 10.1371/journal.pone.0346797

**Published:** 2026-04-13

**Authors:** Deye Ge, Liyan Wu, Jingrong Yang, Jingxian Sun, Jinying Wang, Jingxin Wang, Huihui Song, Ran Wei, Zecheng Xu, Binbin Zhao, Rongfei Sun, Yifei Wang

**Affiliations:** 1 First Clinical Medical College, Shandong University of Traditional Chinese Medicine, Jinan, Shandong, China; 2 Affiliated Hospital of Shandong University of Traditional Chinese Medicine, Jinan, China; 3 The Second Affiliated Hospital of Shandong University of Traditional Chinese Medicine, Jinan, China; 4 Infection Prevention and Control (IPC) Department, Affiliated Hospital of Shandong University of Traditional Chinese Medicine, Jinan, China; 5 Institute of Gerontology, Hubei University of Chinese Medicine, Wuhan, China; 6 Heart Center, Shandong Public Health Clinical Center, Jinan, China; Dow University of Health Sciences, PAKISTAN

## Abstract

The U.S. Food and Drug Administration (FDA) approved intravenous edaravone for the treatment of amyotrophic lateral sclerosis (ALS) in 2017, followed by the approval of the oral formulation in 2022. This study aims to utilize the FDA#39;s Adverse Event Reporting System (FAERS) to investigate the spectrum and timing of adverse events (AEs) associated with edaravone administration, employing repeatability analysis, the Reporting Odds Ratio (ROR) approach, Weibull distribution, and stratification methods. The investigation focuses on data collected from the first quarter of 2017 through the fourth quarter of 2024, aiming to identify adverse event signals and their temporal patterns related to both intravenous and oral edaravone administration. In total, 3,262 records of edaravone-related adverse reactions were identified; among these, 1,534 incidents were associated with intravenous administration, while 453 incidents pertained to oral administration. The analysis revealed distinct adverse reaction profiles for the two routes of administration. Notably, the spectrum of adverse reactions resulting from oral administration predominantly involved the respiratory system, digestive system, and skin damage. In contrast, intravenous administration was more frequently linked to complications associated with invasive procedures and local tissue damage. Furthermore, the timing of adverse reactions exhibited significant variability between the two routes. Weibull distribution analysis indicated that the median onset time for adverse reactions following intravenous administration was 35 days, whereas for oral administration, it was 27 days. Both analytical approaches identified early failure signals, suggesting that the risk of adverse events diminishes over time.

## Introduction

Edaravone is an antioxidant that has demonstrated efficacy in improving outcomes for ischemic stroke and inhibiting delayed neuronal death by scavenging free radicals, including hydroxyl, peroxyl, and superoxide radicals, while also reducing brain edema [1–3]. It was first approved in Japan in 2001 for the treatment of acute ischemic stroke (AIS) via intravenous injection. Subsequently, intravenous edaravone gained approval for use in amyotrophic lateral sclerosis (ALS) in Japan in 2015. The U.S. Food and Drug Administration (FDA) later approved both intravenous and oral formulations of edaravone for ALS in 2017 and 2022, respectively [4]. This progression highlights the growing recognition of edaravone#39;s therapeutic potential in both acute and chronic neurological conditions.

Edaravone is recognized for its effectiveness in treating conditions such as AIS and ALS, yet its potential for significant adverse events (AEs) raises concerns regarding its clinical application [5]. In practice, edaravone can be administered either intravenously or orally, and while both routes are generally regarded as safe [4], the type and frequency of AEs experienced by patients can vary between the two administration methods [6]. This highlights the importance of monitoring patients closely and considering the specific route of administration to optimize the balance between therapeutic benefits and potential risks.

Studies on the intravenous administration of edaravone have identified several common adverse reactions, including allergic reactions [7], mild infusion site infections [8], and hypersensitivity reactions [9,10]. Additionally, Genge [6] suggested that some adverse reactions may be associated with the progression of ALS itself. Moreover, long-term infusion of edaravone carries risks of thrombosis and embolism, which raises further considerations for patient management [5].

Oral edaravone presents a valuable alternative to intravenous administration, alleviating some of the logistical burdens on both patients and healthcare providers [11,12]. In a study evaluating the safety of oral formulations of edaravone, Genge [6] reported that more than 10% of adverse events included falls, muscle weakness, constipation, dyspnea, dysphagia, and back pain.

Current information regarding the adverse reactions associated with edaravone across different routes of administration primarily stems from clinical trials, including cohort studies, retrospective studies, and case-control studies. However, these studies often have strict inclusion criteria and limited sample sizes, which may prevent them from fully capturing the efficacy and safety of the drug across diverse populations [13]. As a result, the actual range of AEs linked to various routes of edaravone administration could be broader than what is currently documented.To bridge this knowledge gap, targeted studies are essential for evaluating the safety of edaravone when administered through different routes. One valuable resource for such research is the FDA Adverse Event Reporting System (FAERS), which is a public database that facilitates the FDA#39;s postmarketing safety surveillance of drugs and therapeutic products. FAERS is one of the largest pharmacovigilance databases globally [14,15] and can provide real-world data that complement findings from clinical trials, helping to establish a more comprehensive understanding of the safety profile of edaravone across diverse patient populations.

This study utilized the FAERS database to conduct a comprehensive examination of the reported potential AEs associated with different routes of edaravone administration, as well as their timing [16]. By employing proportional-imbalance analysis and stratification methods, the research aimed to identify patterns and characteristics of these AEs. For clinicians, understanding the characteristics of AEs associated with different administration routes of edaravone provides valuable references for appreciating the safety correlations linked to its administration. Such insights enhance awareness of safety differences across various administration modes and offer clues to inform subsequent clinical research and risk management of the drug.

## Methods

**Data sources.** This study leveraged the US Food and Drug Administration Adverse Event Reporting System (FAERS) database to systematically evaluate the characteristics of edaravone-related adverse events (AEs) associated with different routes of administration,utilizing both discriminant and stratified analytical methods.The FAERS database is recognized as the world#39;s largest adverse event data reporting system, which aids in understanding drug safety in real-world settings. Through rigorous data mining and analysis, the FAERS database offers valuable insights and authoritative data support for drug safety research [17,18]. For this study, we extracted all reported data related to edaravone from the first quarter of 2017 to the fourth quarter of 2024. This dataset encompasses essential information, including patient demographic details, types of adverse events, medication records, treatment outcomes, reporting sources, and associated diagnoses. By integrating this multi-dimensional data, the study aims to provide a comprehensive analysis of the safety profile of edaravone. The findings will serve as empirical evidence for conducting risk-benefit assessments of the drug, ultimately informing clinical decision-making and enhancing patient safety.

**Fig 1:**
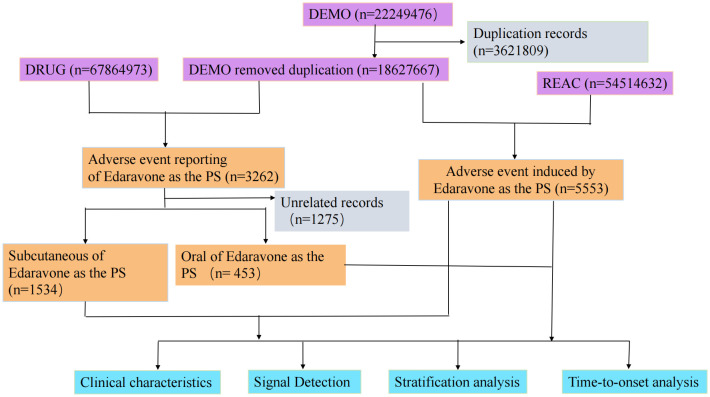
Process of screening edaravone for adverse events associated with different routes of administration from the US Food and Drug Administration adverse event reporting database.

**Data Extraction** and Descriptive Analysis**.** Each report in the FAERS database consisted of orthogonal data on patient demographics (age, sex, country), drug/biologic information (suspect or concomitant medications), AEs, indications for use, and therapy dates [19]. To ensure data uniqueness, we performed a rigorous deduplication process prior to statistical analysis, selecting only reports with the latest FDA acceptance date and removing duplicates using the case ID as the primary key. Records sharing identical primary IDs, patient details, and preferred terms (PTs) within the same case ID were manually excluded, and cases listed in the FDA#39;s quarterly deletion files were subsequently removed [20,21]. Adverse events were coded using MedDRA® (version 25.0) preferred terms, classified into five hierarchical levels (SOC, high-level group term, high-level term, PT, and lowest level term). To improve drug-AE correlation, only records with “role_cod” designated as primary suspected drug were retained. We extracted detailed information including patient characteristics, reporting countries, indications, outcomes, administration routes, time to onset (TTO), concomitant medications, and reporting years. All data processing was performed using MySQL 8.0 (Oracle), Microsoft Excel 2019, and GraphPad Prism 8 (GraphPad Software), with the complete workflow illustrated in Fig 1.

From an initial dataset of 22,249,476 reports which was reduced to 18,627,667 after deduplication, we identified 3,262 related to edaravone adverse event reports through targeted drug screening. As this study focuses on comparing different routes of administration, we excluded 1,275 cases with missing or unspecified routes of administration prior to analysis, and the final analysis included 1,534 intravenous and 453 oral edaravone-related adverse events. This systematic screening process ensures the transparency and reproducibility of our research methods.

### Ethical approval

This article does not contain any studies with human participants or animals performedby any of the authors.

## Results

**Descriptive analysis.** The analysis of edaravone administration data from the FAERS database between Q1 2017 and Q4 2024 reveals important insights regarding patient safety associated with different routes of administration. Across both routes of administration, the AEs related to edaravone were predominantly observed in elderly individuals and male populations. Notably, the risk profiles varied between formulations: intravenous edaravone was associated with higher mortality, whereas the oral formulation showed a stronger correlation with hospitalization.Specifically, we documented 1,534 adverse events related to intravenous edaravone and 453 related to oral edaravone. Our comprehensive analysis aims to explore how these distinct administration routes impact patient safety.The distribution of basic clinical characteristics for patients reporting adverse events is summarized in Table 1. Notably, adverse events associated with intravenous edaravone were more frequently reported in men (45.4%) compared to women (31.1%). Age distribution indicated a significant clustering in the population over 65 years old, which accounted for 21.8% of the total reported events, while the 18–64.9 years age group represented 17.9%.In terms of body weight, individuals in the 50–100 kg category comprised a notable proportion of reports, accounting for 8.7%. Geographically, the United States reported the highest share of adverse events at 68.8%, followed by Japan with 22.7%.Outcomes of these adverse events showed that mortality was the most prevalent outcome, occurring in 36.3% of cases, while hospitalization was noted in 13.4% of the reports.

**Table 1:** clinical characteristics of patients with adverse events under different administration routes of Edaravone.

Characteristics	Intravenous of Edaravone	Oral of Edaravone
X	Overall	Overall
	(N = 1534)	(N = 453)
Sex,n(%)		
Female	477 (31.1%)	155 (34.2%)
Male	696 (45.4%)	204 (45.0%)
Missing	361 (23.5%)	94 (20.8%)
Age(years),n(%)		
<18	4 (0.3%)	22 (4.9%)
18≤and≤65	275 (17.9%)	83 (18.3%)
>65	335 (21.8%)	131 (28.9%)
Missing	920 (60.0%)	217 (47.9%)
Weight(Kg),n(%)		
<50 Kg	23 (1.5%)	11 (2.4%)
50≤and≤100 Kg	134 (8.7%)	29 (6.4%)
>100 Kg	14 (0.9%)	3 (0.7%)
Missing	1363 (88.9%)	410 (90.5%)
Reported countries,n(%)		
US	1055 (68.8%)	224 (49.4%)
JAPAN	348 (22.7%)	187 (41.3%)
Other countries	59 (3.8%)	22 (4.9%)
Missing	72 (4.7%)	20 (4.4%)
Reported,n(%)		
CN	630 (41.1%)	167 (36.9%)
HP	60 (3.9%)	31 (6.8%)
LW	1 (0.1%)	0
MD	456 (29.7%)	171 (37.7%)
OT	258 (16.8%)	26 (5.7%)
PH	127 (8.3%)	58 (12.8%)
Missing	2 (0.1%)	0
Outcomes,n(%)		
DE	557 (36.3%)	110 (24.3%)
DS	6 (0.4%)	0
HO	206 (13.4%)	95 (21.0%)
LT	6 (0.4%)	4 (0.9%)
OT	162 (10.6%)	72 (15.9%)
RI	1 (0.1%)	0
missing	596 (38.9%)	172 (38.0%)
Reporters,n(%)		
Health professional	902 (58.8%)	286 (63.1%)
Consumer	630 (41.1%)	167 (36.9%)
Unknown or missing	2 (0.1%)	0
Characteristics	Intravenous of Edaravone	Oral of Edaravone
Reporting year,n(%)		
2024	71 (4.6％)	130 (28.7％)
2023	89 (5.8％)	142 (31.3％)
2022	93 (6.1％)	47 (10.4％)
2021	94 (6.1％)	13 (2.9％)
2020	204 (13.3％)	22 (4.9％)
2019	272 (17.7％)	24 (5.3％)
2018	601 (39.2％)	53 (11.7％)
2017	107 (7.0％)	21 (4.6％)

AEs, adverse events; n, number of cases.“Missing”denotes incomplete covariates (e.g., gender, age, weight, or reporting country) in cases with confirmed routes.

Similar to the findings for intravenous edaravone, the analysis of oral edaravone administration revealed that adverse events were predominantly reported in men, accounting for 45.0% of cases compared to 34.2% in women. In terms of body weight, the 50–100 kg group was the most represented, comprising 6.4% of the reports. The age distribution highlighted that the group over 65 years old exhibited the highest concentration of AEs, capturing 28.9% of the total, followed by the 18–64.9 years age group at 18.3%.Geographically, the United States reported 49.4% of the adverse events associated with oral edaravone, while Japan accounted for 41.3%. Outcomes associated with these adverse events indicated that mortality was the most common outcome, occurring in 24.3% of cases, followed closely by hospitalization, which was reported in 21.0% of cases. Notably, the increase in reports following the 2022 approval of the oral formulation likely reflects expanded drug availability and clinical utilization rather than a true deterioration in the safety profile.

**Signal intensity of adverse reactions for different routes of administration.** To investigate the differences in AEs between the two routes of administration, we employed the Reporting Odds Ratio(ROR) as our assessment tool. The analysis involved constructing two-by-two contingency tables, as illustrated in Table 2. Positive signals were defined by an ROR value of at least 3 and a lower limit of the 95% Confidence Interval (CI) greater than 1.Using these criteria, we screened for positive signals associated with each route of administration. Subsequently, we ranked the identified AEs in descending order based on their ROR95% CI values. From this ranking, we selected the top 30 adverse events for both oral and intravenous administration for further analysis. The findings of this screening process are summarized in Tables 3 and 4, which provide a detailed overview of the identified positive signals for each route of administration.Oral edaravone was linked to multi-organ damage and severe systemic events, while intravenous administration primarily associated with complications related to invasive procedures and local tissue damage.This observation underscores the necessity for differentiated safety monitoring strategies tailored to each administration route in clinical practice.

**Table 2:** the two-by-two contingency table.

3.2 linked tables in chapters			
	**Target adverse drug event**	**Other adverse drug events**	**Sums**
Edaravone	a	b	a + b
Other drugs	c	d	c + d
	a + c	b + d	a + b + c + d
**3.3 Linked Tables in Chapters**			
	**Target adverse drug event with Edaravone**	**Other adverse drug events with Edaravone**	**Sums**
Oral	a	b	a + b
Intravenous	c	d	c + d
Sums	a + c	b + d	a + b + c + d

**Table 3:** AE signal intensity for oral administration.

	PT	a	ROR	ROR (95%Cl)
Oral	Gastric fistula	4	618.94	618.94 (230.6 - 1661.25)
	Gastrostomy	10	445.79	445.79 (238.5 - 833.24)
	Sputum retention	3	209.65	209.65 (67.36 - 652.57)
	Mechanical ventilation	5	162.48	162.48 (67.39 - 391.75)
	Pneumonia aspiration	10	25.89	25.89 (13.88 - 48.28)
	Subarachnoid haemorrhage	3	18.44	18.44 (5.94 - 57.3)
	Fracture	5	16.82	16.82 (6.98 - 40.51)
	Cerebral haemorrhage	9	16.02	16.02 (8.31 - 30.89)
	Hepatic function abnormal	9	15.89	15.89 (8.24 - 30.65)
	Respiratory failure	12	10.49	10.49 (5.94 - 18.54)
	Choking	3	9.96	9.96 (3.21 - 30.95)
	Retching	3	9.09	9.09 (2.93 - 28.23)
	Jaundice	3	6.92	6.92 (2.23 - 21.5)
	Respiratory distress	3	6.91	6.91 (2.22 - 21.45)
	Respiratory disorder	3	6.4	6.4 (2.06 - 19.89)
	Speech disorder	5	6.02	6.02 (2.5 - 14.49)
	Dysarthria	3	5.04	5.04 (1.62 - 15.65)
	Depressed mood	4	4.95	4.95 (1.85 - 13.21)
	Septic shock	3	4.53	4.53 (1.46 - 14.06)
	Dehydration	9	4.29	4.29 (2.23 - 8.27)
	Nephrolithiasis	3	4.27	4.27 (1.37 - 13.25)
	Productive cough	3	4.2	4.2 (1.35 - 13.03)
	Renal disorder	3	4.09	4.09 (1.32 - 12.69)
	Interstitial lung disease	3	4.04	4.04 (1.3 - 12.56)
	Skin exfoliation	5	3.97	3.97 (1.65 - 9.56)
	Renal impairment	5	3.87	3.87 (1.61 - 9.33)
	Therapy cessation	3	3.8	3.8 (1.22 - 11.79)
	Pneumonia	17	3.44	3.44 (2.13 - 5.56)
	Dysgeusia	4	3.31	3.31 (1.24 - 8.83)
	Dyspnoea	26	2.98	2.98 (2.02 - 4.4)

Note: PT, preferred term; a, number of cases with available; ROR, reporting odds ratio.

**Table 4:** AE signal intensity for intravenous administration.

	PT	a	ROR	ROR (95%Cl)
Intravenous	Cystatin c increased	3	734.58	734.58 (231.96-2326.29)
	Mechanical ventilation	11	125.01	125.01 (69.02 - 226.41)
	Catheter site swelling	4	104.75	104.75 (39.18 - 280.01)
	Haemorrhagic cerebral infarction	3	90.44	90.44 (29.08 - 281.32)
	Gastrointestinal tube insertion	8	74.88	74.88 (37.36 - 150.08)
	Device connection issue	5	69.96	69.96 (29.05 - 168.48)
	Injection site infection	7	47.28	47.28 (22.5 - 99.36)
	Muscle contractions involuntary	6	37.03	37.03 (16.61 - 82.55)
	Central venous catheterisation	5	36.69	36.69 (15.25 - 88.3)
	Catheter site pain	3	28.24	28.24 (9.09 - 87.68)
	Product leakage	5	24.52	24.52 (10.19 - 58.99)
	Catheter site infection	4	24.02	24.02 (9 - 64.1)
	Aphasia	26	18.98	18.98 (12.9 - 27.94)
	Pneumonia aspiration	21	18.93	18.93 (12.32 - 29.09)
	Respiratory failure	49	15.04	15.04 (11.34 - 19.95)
	Infusion site extravasation	4	14.17	14.17 (5.31 - 37.8)
	Device occlusion	8	13.86	13.86 (6.92 - 27.76)
	Dysgraphia	4	13.15	13.15 (4.93 - 35.09)
	Subarachnoid haemorrhage	5	10.72	10.72 (4.46 - 25.77)
	Myasthenia gravis	3	9.57	9.57 (3.08 - 29.7)
	Hepatic function abnormal	14	8.59	8.59 (5.08 - 14.53)
	Death	296	8.57	8.57 (7.6 - 9.67)
	Aspiration	4	8.38	8.38 (3.14 - 22.36)
	Respiratory disorder	11	8.2	8.2 (4.54 - 14.83)
	Vasculitis	4	7.63	7.63 (2.86 - 20.34)
	Cerebral haemorrhage	12	7.42	7.42 (4.21 - 13.08)
	Muscle atrophy	4	7.38	7.38 (2.77 - 19.69)
	Acute respiratory failure	5	5.95	5.95 (2.48 - 14.32)
	Infusion site pain	3	5.57	5.57 (1.8 - 17.3)
	Dysphagia	22	5.25	5.25 (3.45 - 7.99)

Note: PT, preferred term; a, number of cases with available; ROR, reporting odds ratio.

From Tables 3 and 4, it can be observed that there are notable differences in the propensity to induce AEs across various routes of administration. Specifically, for oral administration, the ranked AEs include respiratory failure, Dyspnoea,respiratory distress, respiratory disorder, productive cough, and skin exfoliation. Warnings regarding these adverse effects are included in the product labeling. Additionally, 24 new adverse effects have been identified, including gastric fistula, gastrostomy, mechanical ventilation, pneumonia aspiration, subarachnoid hemorrhage, fracture, cerebral hemorrhage, hepatic function abnormality, choking, retching, jaundice, speech disorder, dysarthria, depressed mood, septic shock, dehydration, nephrolithiasis, renal disorder, interstitial lung disease, renal impairment, therapy cessation, pneumonia, and dysgeusia.

For intravenous edaravone,the most significant adverse events (AEs) included respiratory failure,respiratory disorder and acute respiratory failure, all of which were clearly indicated in the product labeling.Furthermore, during the study,27 new adverse effects were identified,which include: cystatin C increased,mechanical ventilation,catheter site swelling,haemorrhagic cerebral infarction,gastrointestinal tube insertion,device connection issue,injection site infection,muscle contractions involuntary,central venous catheterisation,catheter site pain,product leakage,catheter site infection,aphasia,pneumonia aspiration,infusion site extravasation,device occlusion,dysgraphia,subarachnoid haemorrhage,myasthenia gravis,hepatic function abnormal,death,aspiration,vasculitis,cerebral haemorrhage,muscle atrophy,infusion site pain,dysphagia.

The comparative analysis indicates significant differences in the propensity for AEs induced by different routes of edaravone administration.The spectrum of adverse reactions associated with oral administration primarily involves issues related to the respiratory system, digestive system, and skin damage.Notably, among the 24 newly identified adverse reactions, several stand out:Gastric Fistula,Choking,Digestive tract symptoms such as Dysgeusia.These findings suggest that oral administration may be linked to multi-organ damage, including hepatic function abnormalities, renal impairment, and interstitial lung disease,potentially reflecting direct stimulation of the gastrointestinal mucosa or systemic metabolic disturbances.It is particularly concerning that a higher proportion of new AEs in the oral group were classified as severe, including:Septic Shock,Subarachnoid Hemorrhage,Therapy Cessation.These severe adverse events underscore the need for heightened vigilance regarding potentially fatal risks that may not be adequately covered in the product labeling. In contrast, the intravenous route appears to be more prone to complications related to invasive procedures and local tissue damage. In addition to the respiratory issues already listed in the product label (such as acute respiratory failure), our analysis identified 27 newly discovered adverse reactions associated with intravenous administration.Significant local reactions include: Catheter Site Swelling,Injection Site Infection,Infusion Site Extravasation.Moreover, risks directly associated with device operation, such as issues related to device connection and central venous catheterization, were not addressed in the existing instructions.This oversight highlights the importance of comprehensive risk communication for healthcare providers to ensure patient safety during intravenous administration.

**Fig 2:**
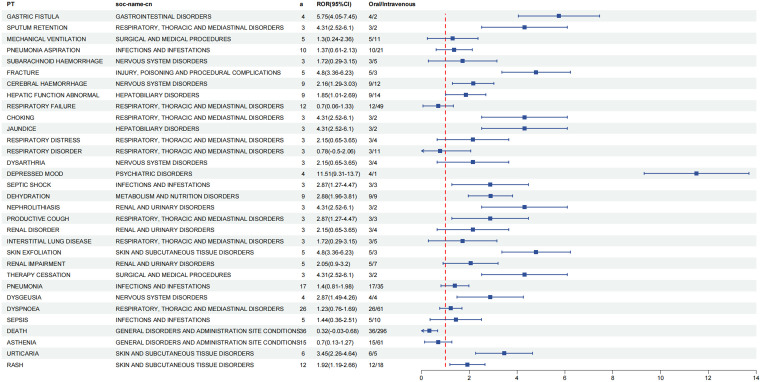
Analysis of differential risk signals for different administration routes of Edaravone. (A)CI, confidence interval;(B)ROR, reporting odds ratio; (C)a, number of cases with suspected AEs associated with the target drug.

**Distributional characteristics of common adverse reactions.** This study aimed to investigate whether the common adverse effects associated with edaravone are more prevalent with intravenous or oral administration routes.Our study indicates that oral edaravone was associated with a significantly broader spectrum of adverse reactions, while intravenous administration was significantly linked only to an increased risk of death.We conducted a Reporting Odds Ratio (ROR) analysis, a multivariate technique, on relevant studies. Table 2 presents the quadratic binomial contingency tables, in which we organized the data based on the frequency of adverse events. We selected the first 32 adverse events and ranked them in descending order according to their ROR 95% CI values. The findings are illustrated in Fig 2.Positive signals for adverse reactions were identified by using ROR 95% CI values. Specifically, an adverse effect is deemed more likely to occur with oral administration if the ROR exceeds 1 and the confidence interval does not include 1. Based on these criteria, we identified several adverse effects that were more frequently associated with oral administration:gastric fistula,sputum retention, fracture,cerebral haemorrhage, hepatic function abnormal,choking,jaundice,depressed mood, septic shock, dehydration, nephrolithiasis, productive cough, skin exfoliation, therapy cessation,dysgeusia,urticaria,rash.Conversely, an ROR of less than 1, along with a CI that excludes 1, suggests that an adverse effect is more likely to occur with intravenous administration. Notably, the only adverse effect identified as more frequent with intravenous administration was death.

**Fig 3:**
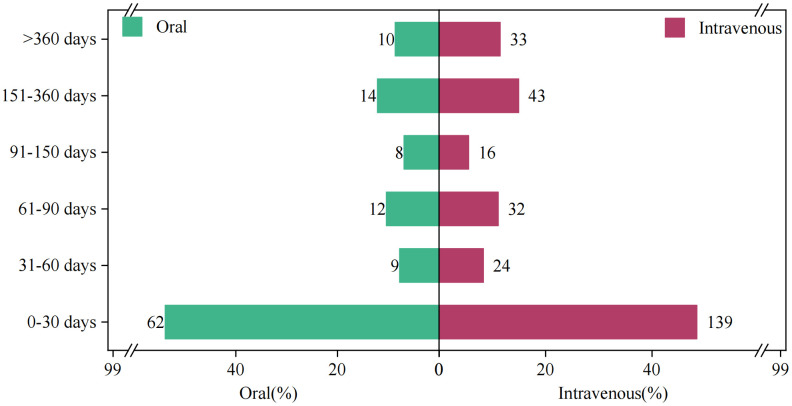
The induction time of adverse reactions associated with different routes of administration.

**Induction time of relevant adverse reactions under different routes of administration.** By analyzing the timing data of adverse effects associated with edaravone in the FAERS database, we created Fig 3 to illustrate the incidence of adverse events (AEs) by different routes of administration. Our research indicates that the incidence of adverse reactions during the first month of oral edaravone administration was significantly higher than that associated with intravenous administration. However, the risk of adverse reactions was lower for long-term treatment (beyond 360 days). Notably, most AEs occurred within the first month and diminished over time, particularly within the first 180 days, for both administration methods.Comparative analysis revealed that, compared to subcutaneous injection, oral administration of edaravone significantly increased the incidence of adverse reactions in the first month (from 48.43% to 53.91%). Regardless of the administration route, the majority of adverse events occurred within the first month of use, with 53.91% for oral edaravone and 48.43% for subcutaneous edaravone. Furthermore, the rates of adverse events were significantly lower in patients treated with oral edaravone for more than 360 days, decreasing from 11.50% to 8.70% compared to subcutaneous dosing. Throughout the remainder of the treatment period, few differences in the probability of adverse events were observed between the two routes. Overall, treatment with oral and intravenous edaravone during the first 180 days generally showed a gradual reduction in the incidence of adverse effects over time.

**Time-to-onset analysis.** Table 5 presents the results of the onset time and weighted signal proportion (WSP) analyses for clinically preferred signals of edaravone AEs associated with both intravenous and oral administration.The analysis revealed that the median time to the occurrence of AEs was shorter for oral administration compared to intravenous administration. However, both routes exhibited an early failure pattern, characterized by a shape parameter β < 1, indicating a significant gradual reduction in the risk of AEs with prolonged treatment duration.The median time to the occurrence of AEs was 35 days (interquartile range [IQR]: 7–158 days) for intravenous administration, compared to 27 days (IQR: 7–99 days) for oral administration. Notably, in the WSP analysis, the shape parameter β and the upper limit of its 95% confidence interval (CI) were both less than 1 for both routes of administration. This finding suggests that the clinical preference signals for both intravenous and oral administration are indicative of early failure, implying a gradual reduction in the risk of AEs over time.

**Table 5:** The analysis of the onset time of priority signals for intravenous injection and oral administration.

Prioritization				Weilbull distribution				Failure type
	Case	TTO (days)		Scale parameter		Shape parameter		
	n	Median (IQR)	Min-max	α	95%CL	β	95%CL	
Intravenous	287	35 (7-158)	1-1722	82.84	65.16-100.52	0.57	0.52-0.63	Early failure
Oral	115	27 (7-99)	1-1722	67.55	44.56-90.55	0.57	0.49-0.65	Early failure

Note: n, number of cases with available time-to-onset; IQR, interquartile range; TTO, Time-to-onset.

## Disscusion

While we acknowledge that FAERS is publicly accessible, our contribution lies in the unique comparative framework rather than the data source itself. Prior pharmacovigilance studies have exclusively examined intravenous edaravone in isolation; to our knowledge, this represents the first large-scale real-world comparison of the safety profiles between oral and intravenous formulations. Our findings regarding intravenous edaravone align with previous clinical trials and literature reviews that identified anaphylaxis and infusion site infections as common adverse effects associated with this administration route [7]. We provide a comprehensive characterization of oral formulation-specific AEs, specifically identifying respiratory complications, skin exfoliation, and mucosal damage signals that have not been systematically documented.Multi-dimensional stratification by age, gender, body weight, and reporter source reveals vulnerable subpopulations including elderly patients who may exhibit differential susceptibility to route-specific toxicities. The divergence in organ-specific toxicities between administration routes suggests distinct mechanistic pathways, with gastrointestinal and respiratory events predominating in oral administration compared to localized infusion reactions with intravenous use. These findings provide crucial directional insights for subsequent pharmacovigilance activities and targeted clinical monitoring as oral formulations gain wider adoption.

Through demographic analysis, we found that oral administration of edaravone was associated with a higher incidence of adverse events in women, while it was linked to fewer adverse events in men compared to intravenous administration. However, the overall proportion of adverse events was greater in men than in women for both oral and intravenous routes. This finding suggests that males may be more sensitive to edaravone than females. Additionally, relevant studies indicate that female hormones, such as progesterone and estrogen, may offer a protective effect against the etiology of amyotrophic lateral sclerosis (ALS) by inhibiting C9orf72 amplification [22]. This hormonal influence may contribute to the higher incidence of ALS observed in males. Consequently, the use of edaravone among the male population has increased, resulting in a larger cohort of male patients in our study. For patients of all ages, the incidence of adverse reactions associated with oral administration was found to be higher than that associated with intravenous administration. Notably, the increased risk of adverse events in older patients (aged 65 and older) may be influenced by long-term use of oral medications to manage chronic conditions. It is important to emphasize that older patients were more likely to experience adverse events with both routes of administration. This trend may be attributed to the rising incidence of ALS with age, which peaks between 60 and 79 years [23,24]. Therefore, we advocate for close monitoring of adverse events, particularly in elderly male patients, as these events can be potentially fatal or accelerate disease progression and must be identified promptly. Our findings revealed that oral administration was associated with a higher proportion of hospitalization-requiring adverse reactions, whereas intravenous administration demonstrated a higher proportion of severe outcomes, specifically death and disability.

The median time to onset of adverse reactions following edaravone treatment was 35 days, with most events (163 cases, 56.8%) occurring within the first two months. For oral administration specifically, the median time to adverse reactions was shorter, at 27 days, with the majority (62 cases, 53.9%) occurring within the first month. Compared to intravenous administration, oral administration significantly accelerated the time to the occurrence of adverse reactions. This phenomenon may be attributed to the longer half-life of oral edaravone compared to intravenous administration [25]. Consequently, these findings suggest that to maximize patient safety, special attention should be given to monitoring for edaravone-related adverse events during the first month of treatment for patients receiving oral administration, while extended observation may be warranted for those receiving intravenous administration.

Allergic reactions and infections at the infusion site are the most common adverse effects associated with intravenous edaravone administration. These adverse effects include infusion site extravasation, infusion site pain, injection site infection, catheter site swelling, catheter site pain, and catheter site infection. Additionally, risks directly related to device operation, such as product leakage, device occlusion, and device connection issues, should not be overlooked. This observation is consistent with findings reported by Witzel [7]. However, the FAERS database lacks procedural details (e.g., catheter types, infusion protocols) that could help distinguish drug toxicity from device-related complications. Consequently, these events likely reflect the technical associated with intravenous delivery rather than intrinsic drug effects, warranting prospective validation with standardized protocols.Early symptoms of ALS include muscle weakness, muscle cramps, and slurred speech [12]. As the disease advances, patients may experience respiratory failure and dysphagia. Notably, respiratory failure is the leading cause of death in ALS [26]. In cases of severe respiratory dysfunction, some patients may require mechanical ventilation.Dysphagia can lead to complications such as central venous catheterization and pneumonia aspiration, with some patients opting for gastrointestinal tube insertion [27]. In our signal mining analysis, cystatin C increased (ROR = 734.58) emerged as the adverse event signal with the strongest value, highlighting the importance of closely monitoring renal function during edaravone treatment.Given that elevated cystatin C levels have been associated with an increased risk of cardiovascular disease [28], extra caution is warranted when administering intravenous edaravone to patients with a history of cardiovascular issues.Feng [29] conducted a systematic review and meta-analysis of randomized controlled trials involving edaravone for treating acute cerebral hemorrhage, indicating that edaravone may cause common adverse reactions, including liver and kidney damage and arrhythmia. These findings are consistent with previous studies, emphasizing the need for clinicians to closely monitor liver and kidney function in patients receiving intravenous edaravone and to conduct a comprehensive assessment of cardiovascular risk. Moreover, a retrospective study of 76 patients treated with edaravone within 24 hours after an acute cardiogenic embolism found that edaravone was associated with an increased frequency of hemorrhagic transformation [30]. In light of these findings, clinicians should be particularly vigilant regarding the risks of hemorrhagic cerebral infarction, subarachnoid hemorrhage, and cerebral hemorrhage during the clinical use of edaravone. However, these potential adverse effects require further verification through additional clinical trials.

Gastric fistula (ROR = 618.94) and gastrostomy (ROR = 445.79) have been identified as the most significant adverse event signals associated with the oral administration of edaravone.However, these signals are more likely indicative of the progression of ALS rather than adverse reactions to the drug itself. Dysphagia is a common complication that arises during the progression of ALS [31]. To ensure adequate nutritional support and facilitate drug administration, patients often require gastrostomy procedures such as percutaneous endoscopic gastrostomy (PEG) [32]. Notably, edaravone oral suspension can be administered through PEG tubes [11], and gastric fistula is a known surgical complication following PEG placement [33]. Additionally, hepatic function abnormal have been reported as the most common adverse effect of oral edaravone, occurring in 4.4% of patients, with a higher incidence among those receiving riluzole. This may be related to riluzole#39;s known effects on liver function [4]. Furthermore, oral edaravone has been associated with renal impairment [34], which aligns with our findings. In Japan, edaravone is contraindicated in patients with severe renal insufficiency and should be used with caution in individuals with hepatic insufficiency [35]. The safety of oral edaravone was evaluated in a phase III, global, multicenter safety study involving 185 patients over 48 weeks [6]. By week 24, adverse events were reported in 146 patients (78.9%), with the most common events being muscle weakness, falls, fatigue, back pain, constipation, headache, and dyspnea. At the 48-week mark, 175 patients (94.6%) reported adverse effects, with the most prevalent reactions being falls (22.2%), muscle weakness (21.1%), constipation (17.8%), dyspnea (10.8%), dysphagia (10.3%), and low back pain (10.3%). These findings differ significantly from those of our study. Given the limited clinical use of oral edaravone, its safety profile remains inadequately established, necessitating further verification through large-scale clinical trials and long-term follow-up studies [4]. In this large-scale real-world study, we found that the adverse reactions associated with oral administration primarily affected the respiratory and digestive systems, as well as skin integrity. Among the 24 newly identified adverse reactions, gastrointestinal symptoms such as gastric fistula, choking, and dysgeusia were particularly notable.This suggests that the drug may be associated with multi-organ damage, including hepatic function abnormalities, renal impairment, and interstitial lung disease, potentially related to direct stimulation of the gastrointestinal mucosa or systemic metabolic disturbances.However, there is currently a dearth of supporting research or literature on this issue, underscoring the necessity for caution.

For patients with ALS, the accessibility of oral therapy and the ability to manage and sustain this treatment are often preferred over intravenous delivery [4]. Many patients expressed dissatisfaction with injections or infusions due to the increased frequency of office visits required for administration and monitoring of potential injection site reactions and other specific side effects associated with these routes. In contrast, administering medications orally or via a nasogastric tube can enhance the quality of life for both patients and healthcare providers [12].

Our research has significant clinical significance.The administration of edaravone through various routes may lead to different adverse effects, which have important implications for both physician and patient safety.For physicians, it is crucial to ensure proper handling of the injection device, particularly for subcutaneous administration, as strict adherence to aseptic techniques is necessary to minimize the risk of infection.Moreover, physicians must be thoroughly informed about the distinct characteristics and potential adverse effects associated with edaravone administered through various routes. This knowledge enables them to promptly identify and manage any adverse reactions that patients may experience, thereby ensuring patient safety. For patients, it is essential to accurately follow physician instructions when administering edaravone through different routes, ensuring that the correct dose and timing are strictly adhered to. By doing so, patients can help mitigate the risk of adverse effects and enhance the overall effectiveness of their treatment.At the same time, patients should be aware that oral administration of edaravone is associated with respiratory and digestive system issues, as well as skin damage. In contrast, subcutaneous injection is associated with a higher risk of complications related to invasive procedures and local tissue damage. Understanding these differences enables patients to monitor their body#39;s responses closely and promptly detect and address any potential adverse reactions. In the event of serious adverse reactions, patients should immediately inform their healthcare providers or seek medical assistance to ensure their safety. For elderly patients with mobility issues, paralysis, or those who are bedridden, it is particularly important to consider physician assistance or to provide training for family members on proper injection techniques. This precaution aims to minimize the risk of injection errors, reduce the likelihood of infection, and mitigate potential adverse effects. By implementing these measures, the accuracy and safety of the injection process can be enhanced, ultimately protecting the patient from possible adverse effects.

While real-world data mining strategies utilizing FAERS databases offer significant advantages for research, it is essential to recognize the inherent limitations of all pharmacovigilance databases, including FAERS. First, the FAERS database employed in this study is based on a voluntary reporting system, which carries inherent risks of reporting bias and underreporting.The collection of information relies on active reporting by medical staff and patients, which can lead to false reports, missing demographic and outcome data that further undermine the validity of subgroup and route-specific analyses, as well as recording errors and inadequate reporting timeliness. All these factors may impair the integrity and reliability of the study data.Second, the reports of deaths and hospitalizations included in our study should be interpreted with caution. ALS is a rapidly progressive and fatal disease, and death and hospitalization may result primarily from disease progression, adverse events, or a combination of both. The limitations of the FAERS database hinder the establishment of causality,as we are unable to determine whether patient deaths or hospitalizations resulted from disease progression or drug administration. We have documented these Oral administration was associated with a higher proportion of cases to facilitate further in-depth investigations in subsequent studies.. Third, there are limitations regarding the representativeness of the data. Most reported cases originate from regions with advanced medical resources, which can be influenced by drug safety events, such as post-marketing surveillance of new drugs and media attention during specific periods. This can lead to selective bias in both time and space, necessitating caution when generalizing the study results to the broader population. External validation through prospective studies is essential. Finally, there are methodological limitations related to study design. Current analyses that focus on the effects of the route of administration do not adequately account for critical variables such as dose adjustments, variations in treatment courses, and patient compliance. These uncontrolled confounding factors may significantly impact the results.

## Conclusion

In conclusion, the adverse effects and their duration associated with edaravone can vary depending on the route of administration. While edaravone is widely used in the treatment of AIS and ALS due to its effective scavenging of hydroxyl, peroxyl, and superoxide radicals, clinicians must be fully aware of the potential side effects linked to different dosage forms when selecting the appropriate route of administration. A comprehensive understanding of these variations can optimize edaravone therapy, potentially reducing adverse effects and improving overall treatment outcomes.
